# Patient and ward related risk factors in a multi-ward nosocomial outbreak of COVID-19: Outbreak investigation and matched case–control study

**DOI:** 10.1186/s13756-023-01215-1

**Published:** 2023-03-22

**Authors:** Jenine Leal, Heidi M. O’Grady, Logan Armstrong, Devika Dixit, Zoha Khawaja, Kate Snedeker, Jennifer Ellison, Joyce Erebor, Peter Jamieson, Amanda Weiss, Daniel Salcedo, Kimberley Roberts, Karen Wiens, Matthew A. Croxen, Byron M. Berenger, Kanti Pabbaraju, Yi-Chan Lin, David Evans, John M. Conly

**Affiliations:** 1 Infection Prevention and Control, Alberta Health Services, Calgary, AB Canada; 2 Department of Pediatrics, Division of Infectious Diseases, University of Calgary and Alberta Health Services, Calgary, AB Canada; 3 Department of Medicine, Cumming School of Medicine, University of Calgary and Alberta Health Services, Calgary, AB Canada; 4 Provincial Population and Public Health, Alberta Health Services, Calgary, AB Canada; 5 Department of Family Medicine, University of Calgary and Alberta Health Services, Calgary, AB Canada; 6 Cardiac Sciences, Foothills Medical Centre, Alberta Health Services, Calgary, AB Canada; 7 Department of Community Health Sciences, Cumming School of Medicine, University of Calgary, Calgary, AB Canada; 8 O’Brien Institute for Public Health, University of Calgary and Alberta Health Services, Calgary, AB Canada; 9 Department of Microbiology, Immunology and Infectious Diseases, University of Calgary, Calgary, AB Canada; 10 Department of Pathology and Laboratory Medicine, University of Calgary and Alberta Health Services, Calgary, AB Canada; 11 Synder Institute for Chronic Diseases, University of Calgary and Alberta Health Services, Calgary, AB Canada; 12 W21C, Department of Medicine, University of Calgary, Calgary, AB Canada; 13 Alberta Public Health Laboratory, Alberta Precision Laboratories, Calgary, AB Canada; 14 Li Ka Shing Institute of Virology, University of Alberta, Edmonton, AB Canada; 15 Department of Medical Microbiology and Immunology, University of Alberta, Edmonton, AB Canada; 16 Foothills Medical Centre, AGW5 - Special Services Bldg, 1403 29Th Street Nw, Calgary, AB T2N 2T9 Canada; 17 Department of Laboratory Medicine and Pathology, University of Alberta, Edmonton, AB, Canada; 18 Women and Children’s Health Research Institute, University of Alberta, Edmonton, AB, Canada; 19 Alberta Public Health Laboratory, Alberta Precision Laboratories, Edmonton, AB, Canada; 20 Infection Prevention and Control, Alberta Health Services, Lethbridge, AB, Canada; 21 Infection Prevention and Control, Alberta Health Services, Edmonton, AB, Canada

**Keywords:** COVID-19, Nosocomial, Outbreak, Risk factors, Case–control, Incidence density sampling, Rate ratio, Viral culture, Environment

## Abstract

**Background:**

Risk factors for nosocomial COVID-19 outbreaks continue to evolve. The aim of this study was to investigate a multi-ward nosocomial outbreak of COVID-19 between 1st September and 15th November 2020, occurring in a setting without vaccination for any healthcare workers or patients.

**Methods:**

Outbreak report and retrospective, matched case–control study using incidence density sampling in three cardiac wards in an 1100-bed tertiary teaching hospital in Calgary, Alberta, Canada. Patients were confirmed/probable COVID-19 cases and contemporaneous control patients without COVID-19. COVID-19 outbreak definitions were based on Public Health guidelines. Clinical and environmental specimens were tested by RT-PCR and as applicable quantitative viral cultures and whole genome sequencing were conducted. Controls were inpatients on the cardiac wards during the study period confirmed to be without COVID-19, matched to outbreak cases by time of symptom onset dates, age within ± 15 years and were admitted in hospital for at least 2 days. Demographics, Braden Score, baseline medications, laboratory measures, co-morbidities, and hospitalization characteristics were collected on cases and controls. Univariate and multivariate conditional logistical regression was used to identify independent risk factors for nosocomial COVID-19.

**Results:**

The outbreak involved 42 healthcare workers and 39 patients. The strongest independent risk factor for nosocomial COVID-19 (IRR 3.21, 95% CI 1.47–7.02) was exposure in a multi-bedded room. Of 45 strains successfully sequenced, 44 (97.8%) were B.1.128 and differed from the most common circulating community lineages. SARS-CoV-2 positive cultures were detected in 56.7% (34/60) of clinical and environmental specimens. The multidisciplinary outbreak team observed eleven contributing events to transmission during the outbreak.

**Conclusions:**

Transmission routes of SARS-CoV-2 in hospital outbreaks are complex; however multi-bedded rooms play a significant role in the transmission of SARS-CoV-2.

**Supplementary Information:**

The online version contains supplementary material available at 10.1186/s13756-023-01215-1.

## Background

The risks of nosocomial transmission of viral respiratory infections [1] have been known for many years and have been recognized in the SARS-CoV-2 pandemic [2]. Nosocomial transmission of SARS-CoV-2 has been reported in acute care institutions from many countries, including Canada [3–6] and highlight how rapidly SARS‐CoV‐2 can spread across hospital wards. Previous outbreaks have revealed common themes, including (1) significant disruption of health care services, (2) the need to enhance infection prevention and control (IPC) measures (3) the promotion of a culture that IPC is everyone’s responsibility and (4) that all healthcare workers (HCWs) need vigilance when assessing patients for COVID-19, appropriate donning and doffing of personal protective equipment (PPE), and to ensure appropriate environmental cleaning [7, 8]. However, evidence continues to evolve on the risk factors for nosocomial SARS-CoV-2 infections among hospitalized patients.

We investigated a multi-ward nosocomial outbreak of SARS-CoV-2 beginning in September 2020 with the following objectives: (1) to describe a nosocomial SARS-CoV-2 infection outbreak investigation on three linked cardiac wards in our acute care tertiary hospital and (2) to conduct a matched-case control study to determine ward and patient-related risk factors for nosocomial transmission of SARS-CoV-2 among cardiac patients.

## Methods

### Setting description of hospital and cardiac wards

Our facility is an 1100-bed tertiary teaching hospital in Calgary, Alberta. The three cardiac wards included two medical cardiac wards on the same floor separated by an elevator bank (Ward A and B) and one cardiac intensive care ward (Ward C) two floors above the medical cardiac wards with frequent patient and HCW movement between the wards. There were 294 admissions and 1,991 patient-days per month across the cardiac wards during the fiscal 2020/2021 year. Wards A and B each had six single-bed, six two-bed, and five four-bed rooms, while Ward C had four single-bed, seven two-bed, and one four-bed rooms. As per our provincial healthcare organization policy, universal admission RT-PCR laboratory testing for SARS-CoV-2 was not employed at any time during the pandemic. Universal admission symptom screening was conducted during the pandemic by our provincial healthcare organization using the COVID-19 Symptom Monitoring Tool [9]. All patients were screened at the time of initial presentation for respiratory symptoms, travel, and COVID-19 exposure to quickly identify those who required additional precautions. For all admitted patients, the COVID-19 Symptom Monitoring Tool [9] was completed by nursing staff at least once daily for the duration of the patient’s hospitalization and recorded in the patient’s medical chart. The outbreak was first declared on 19^th^ September 2020 on Wards A and C, 48 h after five epidemiologically linked patients tested positive from SARS-CoV-2 nasopharyngeal (NP) swabs sent on Sept 17–18, 2020. The symptoms of these patients were thought initially to be due solely to their underlying cardiac disease. Then the outbreak was subsequently declared on 30^th^ September 2020 on Ward B. There was limited community transmission during this time period (active cases, 30.7 per 10,000 population) [10].

### Outbreak investigation

#### Case definition and contact tracing

Case definitions for confirmed or probable cases of COVID-19, outbreak and close contact definitions were based on Public Health guidelines (Additional file 1).

#### Data collection for outbreak investigation and response

Baseline pre-existing hospital and cardiac unit infection control measures along with details of the multidisciplinary outbreak response of investigations and control measures that were initiated at the declaration of the outbreak are outlined in Additional file 1. The multidisciplinary outbreak team met regularly until the outbreak subsided and collated investigation findings and general observations into tabular format. Index date for a case was either the date symptoms started or the date of a laboratory confirmation for SARS-CoV-2, whichever came first. Isolation information was collected from the patient’s medical record (electronic [EMR] and paper) and through discussions with the unit manager. The room on the cardiac wards where a patient with COVID-19 was deemed to have acquired the infection (room attribution) was the room where the patient stayed in within five days prior to symptom onset (based on a median incubation time of 5 days for the original Wuhan strain) [11]. Information on room movement and shared bathrooms was collected from the EMR. HCWs linked to the outbreak were interviewed by Workplace Health and Safety (WHS) using a detailed questionnaire similar to the COVID-19 Symptom Monitoring tool [9] used for patients and included additional questions for forward and backwards contact tracing. Visitors to the affected wards were notified and encouraged to be tested in the community via Public Health if symptomatic or exposed to a known case on the wards. Public Health interviewed all visitors who tested positive for contact tracing purposes and symptom ascertainment.

#### Ventilation assessments

Ventilation, measured in air exchanges per hour (AEH) and percentage outside air were assessed on the three wards by Facilities, Maintenance, and Engineering and interpreted relative to the Canadian Safety Association standards for Heating, Ventilation, and Air Conditioning (HVAC) Systems in Health Care Facilities (CSA-Z317.2-15).

#### Laboratory and virological methods

Serial nasopharyngeal swabs and occasionally throat swabs were collected by experienced personnel and tested for SARS-CoV-2 using a validated real-time RT-PCR assay targeting the E gene with internal controls [12] to obtain cycle threshold (Ct) values. Clinical and environmental specimens obtained from consenting patients from the affected wards were sent to the Li Ka Shing Institute for Virology (University of Alberta) for quantitative viral culture testing as per Lin et al. [13] and PCR assays were performed according to methods previously described [12–14]. Environmental samples were obtained from rooms with known positive patients with a focus on high-touch areas including call bells, bedrails, telephones, cellphones, bathroom sites, commodes, and mobile medical equipment such as pulse oximeters or other oxygen monitoring probes. Symptomatic patients or HCWs were tested for SARS-CoV-2 and serial asymptomatic SARS-CoV-2 RT-PCR prevalence testing was done on all inpatients (q2- 5 days) during the outbreak only and was arranged and strongly recommended for HCWs (q5 days) who worked on the outbreak wards in the 14 days prior to and during the outbreak [9].

#### Whole genome sequencing

The full genome of SARS-CoV-2 strains obtained from the NP swabs of HCWs and patients from the cardiac wards was amplified by multiplex PCR according to the ARTIC V1 or V3 with clean up and no dilutions protocols [15–17] using the Resende oligos [18] as 2000-bp amplicons with sequencing done using Oxford Nanopore. Lineages were assigned using pangolin [19].

### Case–control study

#### Study design and population

A retrospective matched case–control study analyzed the medical records of patients implicated in the COVID-19 outbreak and matched patient controls using incidence density sampling of a dynamic population [20, 21] from our hospital between 1st September 2020 and 15th November 2020.

Case patients were defined as admitted inpatients during the study period who were found to have a laboratory-confirmed COVID-19 infection during routine medical care and that were attributed to the cardiac wards as per outbreak protocols (Additional file 1).

Control patients were defined as inpatients present on the cardiac wards during the study period who either tested negative for SARS-CoV-2, regardless of signs or symptoms, after the outbreak was declared, or were presumed negative if they were identified prior to the outbreak declaration. Of the controls, 80% of the individual control patients were discharged after the outbreak was initially declared and therefore had serial asymptomatic testing, all of which were negative. Of the remaining controls, 20% were discharged prior to the start of the outbreak and would only have been tested for SARS-CoV-2 if they presented with symptoms. On clinical review, none of these patients had any symptoms suggestive of COVID-19 during or after their hospitalization, and in addition 71% of this group had RT-PCR tests done pre- and post-discharge which were all negative. The remaining five control patients who were not tested for SARS-CoV-2 had multiple doctors’ visits with no documentation of symptoms. Control patients were matched to cases if the timing of their stay on the outbreak wards overlapped symptom onset dates of the cases, by age within + /− 15 years of the case age and had a minimum hospital admission of at least 2 days. Controls were initially matched in a n:1 ratio with replacement, whereby each case could have a variable number of controls with some controls used as a control for multiple cases [22]. Each case was matched to controls 1:5. Controls were randomly selected for cases that had more than five matched controls.

For cases who had symptom onset during their hospital admission, exposures were examined 7-days prior to their COVID-19 symptom onset date. For cases who had symptom onset after discharge, hospital exposures were examined in the 7-days prior to discharge. For controls, the dates when outbreaks were declared were considered the index date for controls. For controls admitted to Ward A, 19 September was the index date, and for controls on Ward B, 30 September was the index date. Exposures for controls were examined in the 7-days prior to the start of the outbreak on Ward A (19th September) or Ward B (30th September), depending on when the control case was admitted. Controls admitted after the 30th of September outbreak were excluded.

#### Data collection

Data on case and control patients were collected using retrospective chart review of medical records using a standardized data collection instrument. Demographics data, the Braden Score, baseline medications and laboratory measures were collected from the EMR. Laboratory measures were categorized as abnormal if the results fell outside the normal ranges for each measure as defined by laboratory and clinical criteria (Additional file 1). Comorbidities were collected from admissions in the two years prior to the index date for cases and controls from the Discharge Abstract Database (DAD). Hospitalization characteristics were collected from the Admission, Discharge, Transfer database (Additional file 1).

### Statistical analysis

Outbreak attack rates among admitted patients and case-fatality rates were calculated. Descriptive statistics and univariate conditional logistic regression were used to compare variables, with controls weighted inversely proportional to the number times they were matched to a case, to account for the matching with replacement. Statistical significance was set at *p* < 0.05. All significant variables in the univariate analysis were considered for inclusion in the multivariate conditional logistic regression analysis. Where appropriate, a sensitivity analysis for the multivariate regression was performed using different cut-offs depending on the variable. As the cases and controls were matched on time based on symptom onset date, with exposures considered in a fixed time frame prior to index date, the parameters estimated from the logistic regression are interpreted and reported as incidence rate ratios [20, 21]. The analysis was performed using R version 4.1.1 (IBM Corp, Armonk, NY, USA).

## Results

### Outbreak description

**Fig. 1:**
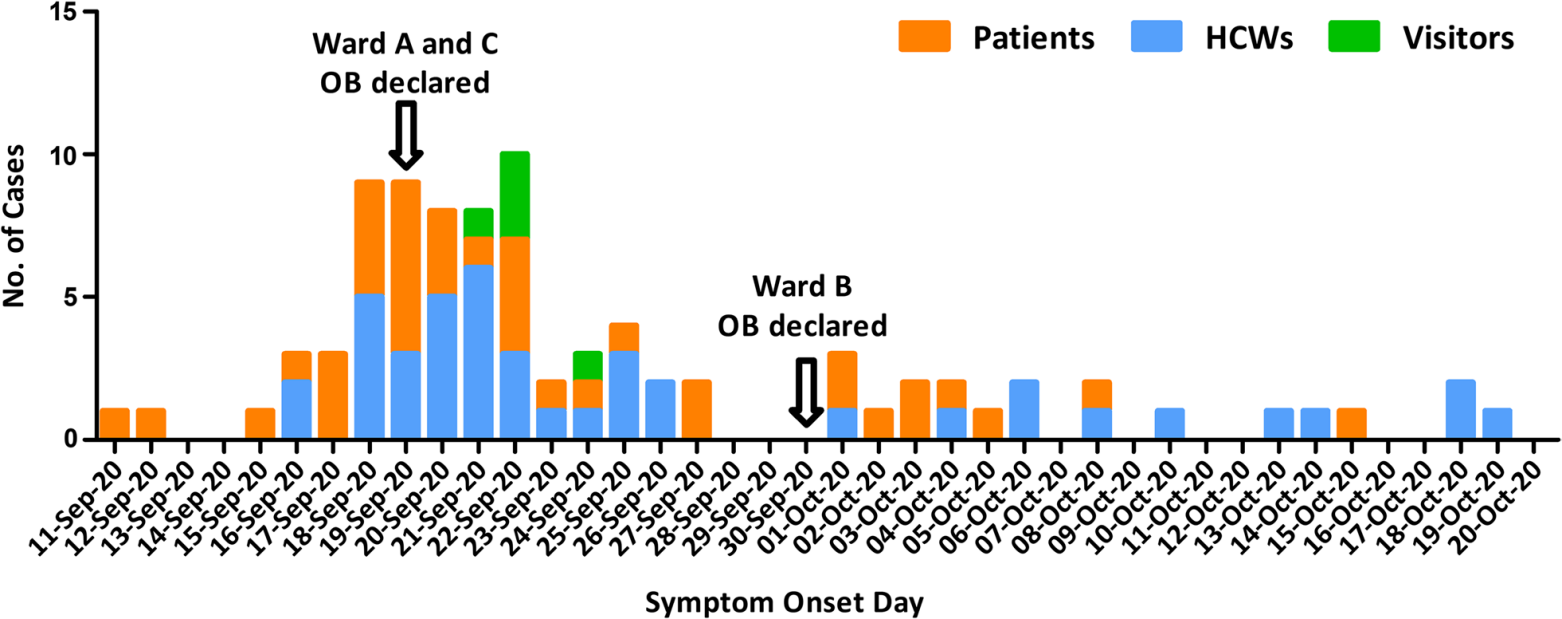
Epidemic curve by index date* for patients across the cardiac wards. Case numbers (Ward: Number): Patients (Ward A:27, Ward C:1, Ward B:11); Healthcare workers, HCWs (Ward A:33, Ward C:0, Ward B:9), and Visitors (Ward A:5, Ward C:0, Ward B:0). *Index date was either the symptom onset date or the date of laboratory confirmation for SARS-CoV-2, whichever came first

The cardiac wards had 685 admissions between 1st September 2020 and 15th November 2020, with an average length of stay of 4.6 days. During the outbreak period, there were 81 cases: 42 HCWs and 39 patients with 10 recorded patient deaths (Fig. 1).

**Fig. 2:**
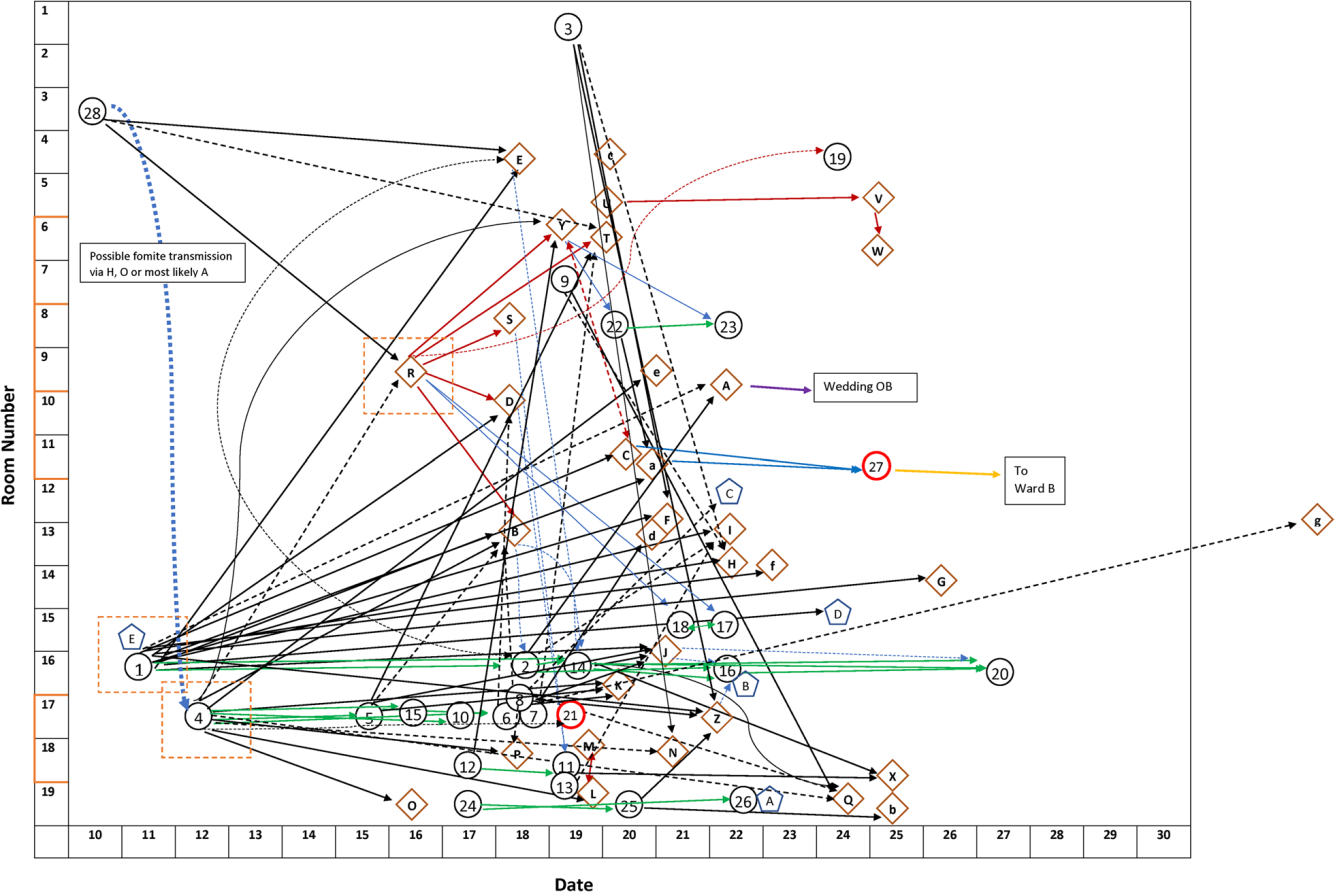
Case Linkage by HCW and Patient Flow Map on Ward A. On 19 September 2020, a COVID-19 outbreak was declared at our facility on Ward A following identification and confirmation of a nosocomial COVID-19 case on Ward A (Patient 28) admitted on 10 September 2020, followed by two patients (Patient 1 and 4) admitted on 11 and 12 September, respectively, that enabled additional transmission events via HCWs to Ward C. Patient 4 was considered to have an exposure from a person visiting the hospital from the community. The outbreak extended to Ward B on 30 September 2020. Black Circle—patient (red outline is a patient transferred to Ward B); Brown Diamond—HCW; Blue Pentagon—visitor. Arrows—transmission pathways (dotted line indicates less likely transmission pathway); Black arrows—patient to staff; Green arrows– patient to patient; Red arrows—HCW to HCW; Orange arrows—to another unit; Blue arrows—HCW to patient; Red dashed line square around patients 1 and 4 and the HCW labelled as R—major nodes of forward transmission

Over half of the patients with COVID-19 were males (56.4%), while HCWs with COVID-19 were mostly females (70.4%). The mean age of patients was 75 years (SD 12), and 38 years (SD 12) among HCWs. The attack rate among patients was 5.7%, with a case fatality rate of 25.6%. All patients and visitors who were found to be SARS-CoV-2 RT-PCR positive were symptomatic, while 40/42 (95.2%) HCWs were found to have symptoms [23]. Although all patients were tested serially while in hospital after the outbreak was declared, testing was not mandatory for HCWs but a total of 1497 RT-PCR tests were collected from 1,011 HCWs, of which 376 HCWs were identified as core nursing and management staff, excluding physicians, residents, allied health professionals, and lab services who were much smaller in number and many of whom were transient on the affected wards. HCW compliance for SARS-CoV-2 prevalence testing was very high during this outbreak given that this was the first major outbreak in our hospital during the pandemic and was in an unvaccinated population. Details of the symptoms in the patients, HCWs and visitors are reported elsewhere [9] and were found in 97.7% of cases, with influenza-like–illness (ILI) symptoms and signs being found in 84.9% of all RT-PCR positive cases. The outbreak network map is provided in Fig. 2.

### Laboratory, virologic, and ventilation results

Of the 73 and 10 pre- and post-cleaning environmental swab samples collected on Ward A 11 (15.1%) and one (10%) were RT-PCR positive for SARS-CoV-2 (*p* = 0.6674) (Additional file 2). On Ward B, 3/55 (5.4%) randomly sampled and 9/13 (69.2%) targeted environmental swabs (stethoscope, pulse oximeter, gown, bedside tables/flooring, urine catheter bag, bedrail, inhaler) were RT-PCR positive for SARS-CoV-2, respectively. There were 64 specimens collected directly from 8 consenting patients, including clinical specimens and their immediate environment from Wards A and B, all of whom had NP Ct values < 20 (N gene; range 11.4–19.2). SARS-CoV-2 was cultured from 34/60 (56.7%) clinical and environmental specimens with titres of ranging between 5.0 × 10^0^ and 5.2 × 10^5^ pfu/ml (Additional file 2: Table A3).

Of the 78 NP specimens collected from both patients and HCWs who were confirmed to be related to the cardiac wards and were successfully sequenced (n = 45), 44 (97.7%) were SARS-CoV-2 lineage B.1.128. Community samples sequenced for SARS-CoV-2 during the same period differed substantially from the outbreak strain with B.1.128 representing only 8.9% of the circulating lineages at the time in our local setting and less than 1% across almost 2000 typed strains across the province at the time.

Ventilation, on Wards A, B, and C ranged from 4.3–10.7, 6.9–14.3, and 10.5–13 AEH, respectively, all with 100% outside air, meeting or exceeding the Canadian Standards Association (CSA) standards of a minimum outdoor AEH of 4 for 100% outside air.

### Sources and contributing events of transmission

The multidisciplinary outbreak team, through its investigations, identified eleven potential sources and contributing events to transmission during this cardiac ward outbreak which are summarized in Table 1.

**Table 1:** Potential sources and contributing events to transmission of SARS-CoV-2 contributing to the outbreak on the cardiac wards

Lapses in routine practices and additional precautions	Failure to isolate symptomatic patients at symptom onset (10 symptomatic patients in hospital were not isolated for a total of 38 days (range 1–10 days) before outbreak were declared)
	Failure to recognize initial cases with illness symptoms compatible with COVID-19 due to crossover with symptoms common to cardiac patients with heart failure (symptomatic patients were not isolated for a total of 15 days (range 1–3) after the outbreaks were declared)
	Inappropriate discontinuation of contact/droplet precautions in five patients identified as close contacts of known cases, who initially tested negative (only to test positive later)
	Premature (prior to 14-day incubation period) discontinuation of contact/droplet precautions
	Suboptimal donning/doffing and hand hygiene by healthcare workers
	Uncertainty around performance of a point-of-care risk assessment
Increased patient-to-patient exposures	Shared rooms and bathrooms among 34 (89.5%) patients leading to close contact between patients and potential transmission events through either respiratory droplets/particles across a continuum of sizes and/or contact (direct or indirect)
	Transfer of seven patients, identified as close contact to other wards resulting in two forward transmission events
Lapses in environmental cleaning	Potential lapses in environmental cleaning leading to fomite transmission
HCW and visitor exposures	Healthcare worker-related transmission events (e.g., shared breakrooms, carpooling, socializing outside of work) and transmission events related to HCWs interacting with patients between wards up until the outbreak was declared and for several days thereafter before cohorting was strictly enforced
	Potential visitor-to-patient transmission with observed visitor non-compliance with masking and distancing recommendations

### Case control study

#### Clinical characteristics of patients

The case control study included all 39 case patients matched to 183 controls, of which 74 were different individuals acting as control patients (Additional file 3). Four controls were excluded based on admission dates resulting in 70 different individual control patients weighted by the number of times they were matched to a case. Table 2 shows demographic data, underlying diseases, laboratory findings and mobility findings seven days prior to the index date for cases and controls. Within the seven days prior to the index date, cases were in hospital longer than controls (median 7 days vs. 4.4 days). The overall median and mean length of stay on the cardiac wards prior to the index date was similar between cases and controls (mean 12.3 vs 13.1 days, median 7.8 vs 6.0 days, respectively). Of the cases, 75.9% (31/39) had underlying chronic diseases (Additional file 3). Compared with the controls, the cases had a higher prevalence of fluid/electrolyte disorders (35.9% vs. 14.3%, *p* = 0.001) and neurological disorders (10.3% vs. 2.9%, *p* = 0.020). Prior to symptom onset, cases were also more likely to have lymphopenia (46.1% vs. 28.6%, *p* = 0.016), were on a diuretic longer (3.03 days vs. 2.41 days, *p* = 0.031) and immunosuppressive agents longer (0.52 days vs. 0.05 days, *p* = 0.003) than controls. Prior to symptom onset, cases were less likely than controls to walk occasionally or frequently (69.2% vs. 85.7%, *p* = 0.0001).

**Table 2:** Patient characteristics prior to index date^a^ during COVID-19 outbreak

Variables	Nosocomial COVID-19 N = 39 (%)^b^	Matched control N = 70 (%)^b^	IRR (95%CI)	*P* value
Age, years, median (IQR)	76 (15.5)	73 (13)	1.04 (1.00–1.07)	0.024
Sex				
Male	23 (59.0%)	49 (70.0%)	0.62 (0.36–1.04)	0.070
Underlying disease^c^				
Other Neurological Disorders	4 (10.3%)	2 (2.9%)	2.04 (1.12–3.74)	0.020
Fluid/Electrolyte Disorders	14 (35.9%)	10 (14.3%)	3.16 (1.57–6.37)	0.001
Elixhauser score (AHRQ)				
Median (IQR)	6 (19.5)	7 (14)	1.03 (-1.05)	0.039
Mean (± SD)	9.87 (11.72)	8.38 (9.44)		
Laboratory findings^d^				
Abnormal WBC count	5 (12.8%)	13 (18.6%)	0.63 (0.31–1.30)	0.212
Abnormal Lymphocyte	18 (46.1%)	20 (28.6%)	1.82 (1.12–2.94)	0.016
Abnormal Creatinine	18 (46.1%)	24 (34.3%)	1.49 (0.94–2.35)	0.087
Abnormal Platelet	5 (12.8%)	7 (10.0%)	1.00 (0.57–1.78)	0.992
Abnormal Hemoglobin	18 (46.1%)	34 (48.6%)	0.86 (0.56–1.32)	0.492
Abnormal Neutrophil	9 (23.1%)	15 (21.4%)	1.01 (0.58–1.74)	0.984
Mobility-slightly limited or no limitations	34 (87.2%)	61 (87.1%)	1.14 (0.54–2.44)	0.726
Activity-walks occasionally or frequently	27 (69.2%)	60 (85.7%)	0.38 (0.23–0.62)	0.0001
Braden score, median (IQR)	20 (18–21)	20 (19–21)	0.97 (0.90–1.05)	0.482
Medications				
Days on ACE inhibitors, mean (SD)	0.82 (1.98)	0.82 (1.85)	0.99 (0.85–1.16)	0.920
Days on angiotensin II inhibitors, mean (SD)	1.05 (2.22)	0.99 (2.20)	1.03 (0.93–1.13)	0.604
Days on angiotensin receptor blockers and neprilysin inhibitors, mean (SD)	0.11 (0.71)	0.06 (0.33)	1.29 (0.78–2.14)	0.321
Days on diuretic, mean (SD)	3.03 (3.16)	2.41 (2.83)	1.09 (1.01–1.18)	0.031
Days on immunosuppressive agents, mean (SD)	0.52 (1.84)	0.05 (0.51)	1.33 (1.10–1.60)	0.003

*ACE* angiotensin-converting enzyme, *AHRQ* Agency for Healthcare Research and Quality, *CI* confidence interval, *IQR* interquartile range, *IRR* incidence rate ratio, *SD* standard deviation

^a^Index date for cases was either the symptom onset date or first laboratory confirmation for SARS-CoV-2, whichever came first. Index date for controls was when the outbreak was declared, either 19 September 2020 or 30 September 2020

^b^Number and percentages displayed, unless otherwise indicated for continuous variables

^c^Only those comorbidities that were significantly different between cases and controls are displayed. Additional file 3 lists results for all comorbidities

^d^Abnormal values based on laboratory measure values falling outside normal ranges as defined by laboratory and clinical criteria

#### Characteristics of hospital stay

Patients who spent more than 50% of their hospital stay in a single-bed room, had a 63% (IRR 0.37, 95% CI 0.15–0.92) lower rate of acquiring COVID-19 in hospital during this outbreak (Table 3), whereas patients who spent more than 50% of their hospital stay in a multi-bedded room had nearly twice the rate of acquiring COVID-19 in hospital (IRR 1.86, 95% CI 1.17–2.94). Specifically, patients that were in multi-bedded rooms between four and seven days were significantly more likely to acquire COVID-19 (IRR 3.89, 95% CI 2.28–6.65) compared to patients who spent less than or equal to two days in a multi-bedded room.

**Table 3:** Characteristics of the hospital stay for cases and controls of the COVID-19 outbreak

Variables	Nosocomial COVID-19 No. (%)	Matched control No. (%)	IRR (95%CI)	P value
Percent of exposure on single room				
0–50% of time	36 (92.3%)	55 (78.6%)	Reference	
> 50% of time	3 (7.7%)	15 (21.43%)	0.37 (0.15–0.92)	0.031
Percent of exposure on double room				
0–50% of time	26 (66.7%)	46 (65.7%)	Reference	
> 50% of time	12 (33.3%)	24 (34.3%)	0.76 (0.44–1.30)	0.310
Percent of exposure on multi-bedded room				
0–50% of time	20 (51.2%)	45 (64.3%)	Reference	
> 50% of time	19 (48.7%)	25 (35.7%)	1.86 (1.17–2.94)	0.008
Days in single room (two-day intervals)				
≥ 0, ≤ 2	35 (89.7%)	57 (81.4%)	Reference	
> 2, ≤ 4	2 (5.1%)	9 (12.9%)	0.44 (0.15–1.31)	0.140
> 4, ≤ 7	2 (5.1%)	4 (5.7%)	0.82 (0.29–2.31)	0.709
Days in double room (two-day intervals)				
≥ 0, ≤ 2	28 (71.8%)	46 (65.7%)	Reference	
> 2, ≤ 4	1 (2.6%)	11 (15.7%)	0.17 (0.02–1.15)	0.069
> 4, ≤ 7	10 (25.6%)	13 (18.6%)	0.74 (0.41–1.31)	0.300
Days in multi-bedded room (two-day intervals)				
≥ 0, ≤ 2	18 (46.2%)	50 (71.4%)	Reference	
> 2, ≤ 4	5 (12.8%)	11 (15.7%)	1.15 (0.58–2.30)	0.686
> 4, ≤ 7	16 (41.0%)	9 (12.9%)	3.89 (2.28–6.65)	0.000

*CI* confidence interval, *IQR* interquartile range, *IRR* incidence rate ratio, *SD* standard deviation

### Patient risk factors for nosocomial COVID-19 outbreak

The multivariate analysis (Table 4) revealed that fluid/electrolyte or neurological disorders, days on immunosuppressive agents, and percent of exposure in a multi-bedded room were independent risk factors for nosocomial COVID-19. A sensitivity analysis for the multivariate regression was performed using different cut-offs (25%, 75%) for percentage of exposure time in multi-bedded rooms. The rate ratio of a nosocomial COVID-19 case increased from 1.89 (95% CI 1.04–3.43) at the > 25% cut-off to 3.32 (95% CI 1.47–7.02) at the > 75% cut-off for percentage of time spent in a multi-bedded room (Additional file 3).

**Table 4:** Independent risk factors for nosocomial COVID-19 from multivariate analysis

Variables	aIRR	95%CI	*P* value
Age	1.04	1.00–1.08	0.073
Underlying disease			
Fluid/electrolyte disorders	3.82	1.62–9.02	0.002
Other neurological disorders	2.66	1.32–5.38	0.006
Medications			
Days on diuretic	1.05	0.95–1.15	0.331
Days on immunosuppressive agents	1.39	1.06–1.83	0.018
Elixhauser score (AHRQ)	0.97	0.93–1.01	0.132
Abnormal lymphocyte	1.38	0.75–2.55	0.302
Percent of exposure on multi-bedded room			
0–50% of time	Reference	Reference	Reference
> 50% of time	3.21	1.47–7.02	0.003

*AHRQ* agency for healthcare research and quality, *CI* confidence interval, *aIRR* adjusted incidence rate ratio

*Age, neurological disorders, fluid/electrolyte disorders, Elixhauser score, lymphocyte levels, activity, days spent on diuretics, days spent on immunosuppressive agents, and time spent on single bed and/or multi-bed rooms from univariate analysis were included in multivariate analysis. Activity was removed due to missing data, and of the variables capturing room use, only the percent of exposure in a multi-bed room was included due to heavy multicollinearity between these variables

### Control measures and interventions

Control measures included but were not limited to: active versus passive fit-for-work screening among HCWs with staff symptom screening and temperature checks twice per shift; continuous masking and eye protection by HCWs; enhanced education on PPE including a PPE Safety Coach Program [24]; ward logs for tracking staff and physicians entering the wards, continuation of asymptomatic testing every five days for as long as the HCW worked on any impacted ward, exclusion of non-essential HCWs (e.g. students, volunteers) on the affected wards; and HCW cohorting on all outbreak wards, aided by a single-site order restricting HCWs to only work on a specific ward without moving between wards. Other control measures included, visitation restrictions, enhanced cleaning of high touch or shared equipment, symptom screening twice daily for patients on all outbreak wards, discontinuation of precautions by the most responsible healthcare practitioners, with IPC approval, use of dedicated bedside commodes in two-bed and multi-bedded rooms with shared toilets and blocking beds to reduce the number of multi-bedded rooms being used.

## Discussion

Our study is one of several observational studies exploring risk factors for patient COVID-19 acquisition in hospitals [25–32]. There were several factors which were identified during this outbreak in a setting without vaccination for any HCWs or patients which may have facilitated the transmission events to occur. Failure to isolate symptomatic patients on symptom onset likely led to transmission via close contact [33] through either respiratory droplets/particles across a continuum of sizes, and/or contact (direct and indirect) routes of transmission within shared rooms/bathrooms. A retrospective cohort study found in a crude analysis of 122 patients across three outbreak wards that being exposed to a symptomatic COVID-19 patient within the same 4-bed bay regardless of proximity in the room was associated with doubling the risk of becoming a case (crude RR, 2.3, 95% CI 1.42–3.65) [27]. In a matched case–control by Aghdassi et al. [28], the multivariate analysis revealed that presence on a ward that experienced a COVID-19 outbreak (aOR 15.9, 95% CI 2.5–100.8) and documented contact with a COVID-19 case (aOR 23.4, 95% CI 4.6–117.7) to be the primary factors for nosocomial COVID-19 infections in patients [28]. This latter study and the results of our outbreak investigation supports the need to preemptively isolate patients known to be exposed to cases.

Based on our findings, patients who spent > 50% of their admission in a multi-bedded room had 3.2 times the rate of acquiring COVID-19. Other observational studies have demonstrated that multi-bedded rooms versus one-to-two bedded rooms and the use of shared toilets were more common among nosocomial COVID-19 cases compared to controls [29, 31, 34, 35]. The duration of time in a multi-bedded room was a major risk factor and the finding of a dose–response relationship adds epidemiologic strength of association to this finding. Another study from Singapore in a large cohort of nosocomial SARS-CoV-2 infections in patients housed in 5–6-bed cubicles, during time periods encompassing both SARS-CoV-2 Delta and Omicron variants found that sharing a common toilet with ≥ 1 cohorted cubicle was an independent risk factor for a transmission event (aOR, 1.92; 95% CI, 1.02–3.62) along with performance of aerosol-generating procedures and a cycle-threshold value of < 20 on RT-PCR testing [36]. This latter finding corroborates our finding of a 3.2-fold increased rate of acquiring SARS-CoV-2 with exposure to a multi-bedded room with a shared toilet and adds additional support given that it is irrespective of variant strain of SARS-CoV-2.

Another outbreak factor was the delayed recognition of initial cases with illness symptoms compatible with COVID-19 due to crossover with symptoms common to cardiac patients with heart failure (shortness of breath, cough, chest pain, dyspnea). It was difficult to know if clinical judgement, situational factors (e.g. staffing shortages, workarounds), or compromised HCW psychological and physical safety (e.g. stress, fatigue, burnout) resulted in suboptimal point-of-care risk assessment resulting in missed, delayed, or incorrect diagnosis of COVID-19 among these patients leading to preventable exposures and increased transmission [37]. Precise case identification is essential to isolate vulnerable individuals and hence contain transmission [38, 39].

A surprising finding was that individuals with underlying fluid and electrolyte disorders had nearly four times the rate of acquiring COVID-19. It has been hypothesized that the renin–angiotensin–aldosterone system and its core factor ACE2 which regulates electrolyte homeostasis may play a role in the acquisition of COVID-19 [40, 41]. Dehydration, chronic hypertonicity, and/or hypovolemia before COVID-19 infection can alter levels and/or activities of hormones that depend on cell volume (e.g. insulin) and/or balances total body water (e.g. aldosterone), which increases ACE2 receptors potentially making individuals more susceptible to infection [41]. It is also possible that by virtue of their underlying cardiac conditions, these patients may have had a higher degree of pre-existing fluid and electrolyte disorders.

Many studies have reported on the identification of SARS-CoV-2 RNA on inanimate surfaces; however, some authors have suggested that the risk of transmission of SARS-CoV-2 through fomites is low [42]. A recently published systematic review identified that infectious SARS-CoV-2 is indeed present on fomites in multiple settings, especially high frequency touched surfaces. Infectious SARS-CoV-2 on fomites was significantly more likely when the RT-PCR Ct values for clinical specimens and fomite samples was < 30 and most frequently detected within the first week of symptom onset in immunocompetent individuals [43, 44]. Other studies have corroborated the finding of infectious virus being very strongly correlated with low Ct values, irrespective of the variant [45, 46].

Data presented from our viral cultures showing very high quantitative burdens of infectious virus both from patients and their immediate surroundings in conjunction with very low Ct values lends support that direct and indirect (fomite) transmission played a role as a route of transmission. For this outbreak, the culturable virologic and RT-PCR patient and environmental data would lend support to contact transmission occurring within multi-bedded rooms and/or shared bathrooms, especially in the setting of continuous surgical mask wearing by all HCWs, and ventilation parameters exceeding standards and with 100% outside air. We cannot exclude mixed modes of transmission, but the relative protection provided for nosocomial acquisition by patients within single rooms argues against long range airborne transmission. We cannot exclude poor hand hygiene and suboptimal PPE practices by HCWs which have been implicated previously as associated with nosocomial transmission of SARS-CoV-2 by HCWs, even with the use of full PPE [44, 47].

Our outbreak investigation identified multiple exposures among HCWs from symptomatic patients before they were diagnosed with COVID-19, despite the use of continuous masking by all HCWs but without other components of PPE, which may have contributed to acquisition of COVID-19 among HCWs. Doffing of PPE in the appropriate manner and sequence is critical to prevent self-contamination [48–50].

Our study is not without limitations. The retrospective nature of our case–control study precludes conclusions of causation for acquisition of COVID-19. Nosocomial cases were investigated more thoroughly during the outbreak, whereas data on controls were collected retrospectively. Selection bias is a common limitation of case–control studies; however, we believe this bias was mitigated by selecting controls matching by age with similar clinical health statuses and ensuring controls overlapped their time in hospital with the case patients. Both cases and controls would have been exposed to similar outbreak control measures. Confounding bias may exist. Information on HCWs was insufficient to include in the study. Although we did not employ universal admission RT-PCR testing for SARS-CoV-2 as per local policy, recent recommendations argue against its routine use for asymptomatic persons in healthcare facilities [51].

There have been many reports on hospital-based outbreaks of COVID-19 [4, 25, 27–29, 31, 39, 52–56], however a strength of our report is that it incorporated a case–control study to explore contributing ward and patient-related factors to the acquisition of COVID-19, occurring in a setting without vaccination for any HCWs or patients. Our outbreak has similarities with other COVID-19 nosocomial outbreaks including unidentified cases on a ward [39, 56], positive HCWs who may have sub-optimal adherence to IPC measures [52, 54], and the role of multi-bedded rooms in SARS-CoV-2 transmission [27, 29, 31, 34, 35] Further, our report included patient symptoms, environmental sampling, whole genome sequencing, viral culture and has identified the novel finding of fluid and electrolyte disorders increasing the likelihood of COVID-19 acquisition.

## Conclusion

In conclusion, conducting outbreak investigations and evaluating hypotheses epidemiologically is critical in identifying sources of and measures to mitigate transmission of SARS-CoV-2. Key learnings for future outbreaks include but are not limited to (1) recognizing structural and organizational elements in hospitals i.e. multi-bedded rooms, frequent patient movements, routes of patient movement that may contribute to potential spread and include them in pandemic responses; (2) recognizing the contribution of contaminated surfaces and especially mobile medical equipment; the need for careful cleaning and disinfection; and the need for hand hygiene with compliance monitoring, (3) prompt identification of COVID-19 patients; (4) using consistent approaches to ending contact/droplet precautions; (5) minimizing patient transfers; and (6) maintaining adequate training of HCWs in the principles of infection prevention and control including vigilance in donning and doffing of PPE.

## Supplementary Information

**Additional file 1**. **Definitions**: Lists full definitions for outbreak, case definition, and data sources. It also provides additional details about data collection.

**Additional file 2**. **Environmental sampling results: **Includes three tables of the environmental sampling results before and after cleaning, and cultivatable virus detected from clinical and environmental specimens.

**Additional file 3**. **Additional case-control details and results**: Includes case-control matching details, breakdown of comorbidities between cases and matched controls, and independent risk factors for nosocomial COVID-19 using different cut-off points for the percent exposure on multi-bedded rooms.

## Data Availability

The datasets generated and/or analyzed during the current study are not publicly available so as to not compromise patient identity.

## Abbreviations

ACE2: Angiotensin converting enzyme 2
AEH: Air exchanges per hour
ARECCI: A Project Ethics Community Consensus Initiative
CHREB: Conjoint Health Research Ethics Board
COVID-19: Coronavirus disease 2019
CSA: Canadian Standards Association
Ct: Cycle threshold
DAD: Discharge Abstract Database
EMR: Electronic medical record
HCWs: Healthcare workers
HVAC: Heating, ventilation, and air conditioning
IPC: Infection prevention and control
IRR: Incidence rate ratio
NP: Nasopharyngeal
OR: Odds ratio
ORION: Outbreak Reports and Intervention studies of Nosocomial infection
PPE: Personal protective equipment
RNA: Ribonucleic acid
RT-PCR: Reverse-transcription polymerase chain reaction
SARS-CoV-2: Severe acute respiratory syndrome Coronavirus-2
SD: Standard deviation
WHS: Workplace Health and Safety
