# Supplementary material for: Patient and ward related risk factors in a multi-ward nosocomial outbreak of COVID-19: Outbreak investigation and matched case–control study
Source: Antimicrob Resist Infect Control. 2023 Mar 22;12:21. doi: 10.1186/s13756-023-01215-1 (PMC10031162; doi:10.1186/s13756-023-01215-1)
Supplement: Supplementary file 1 — Additional file 1. Definitions: Lists full definitions for outbreak, case definition, and data sources. It also provides additional details about data collection. [file 13756_2023_1215_MOESM1_ESM.docx]

**ADDITIONAL FILE 1: DEFINITIONS**

**Baseline Hospital and Cardiac Unit Infection Control Measures**

Between February and September 2020, the hospital implemented multiple infection control policies, including the continuous use of medical/surgical masks by all HCWs, provision of additional alcohol hand hygiene dispensers, terminal room cleaning with accelerated hydrogen peroxide, introduction of a patient COVID-19 symptom identification monitoring tool (COVID-19 SIMT) [10], screening on entry to the hospital by visitors and a Fit for Work Screening tool for HCWs with instructions for ill HCWs to stay home. The outbreak response was multidisciplinary involving Site Administration, The Cardiac Unit team, Workplace Health and Safety, Public Health and led by Infection Prevention and Control and was launched on the date the outbreak was declared and concomitantly with the investigations, including use of the COVID-19 SIMT three times daily, forward and backward contact tracing, serial RT-PCR SARS-CoV-2 testing, movement tracking, enhanced cleaning, and closure to new admissions and other measures, details of which can be found elsewhere [1].

**Outbreak Definition**

A COVID-19 outbreak in acute care was defined (according to our local guidelines from 2020; these internal documents are available upon request) as follows:

- One (1) or more confirmed COVID-19 patient case(s) OR
- Two (2) or more confirmed COVID-19 cases in healthcare workers (HCWs) assigned/linked to a ward within a 14-day period AND where at least one of the HCWs was in the workplace during the communicable phase of illness AND/OR it is suspected there has been work site transmission as cause for one or more of the infections.

**Case Definition**

Cases of COVID-19 were included if they were confirmed or probable cases according to the provincial case definitions in August 2020 (See the [Alberta Public Health Disease Management Guidelines: Coronavirus – COVID-19](https://open.alberta.ca/dataset/a86d7a85-ce89-4e1c-9ec6-d1179674988f/resource/fbf3906c-0ebe-462d-bb23-0f2bbaa7598c/download/covid-19-guidelines-2020-08-28.pdf)).

**Confirmed case:**  A person with laboratory confirmation of infection with the virus (SARS-CoV-2) that causes COVID-19 which consists of detection of at least one specific gene target by nucleic acid amplification tests (NAAT) at any laboratory within Alberta Precision Laboratories where NAAT tests have been validated or a confirmed positive result by the National Microbiology Lab (NML) by NAAT.

**Probable case:** A person (with no laboratory testing done) with:

1. clinical illness who had close contact to a laboratory-confirmed COVID-19 case OR
2. a person (with laboratory testing done) with clinical illness who meets the COVID-19 exposure criteria AND in whom laboratory diagnosis of COVID-19 is inconclusive.

**Primary outbreak case:**  A confirmed or probable case that was also epidemiologically linked to the facility (where the most likely source of infection was an exposure at the outbreak unit and the individual was exposed within 14 days prior to symptom onset).

**Secondary or tertiary outbreak case:** A confirmed or probable case with a household contact or other secondary exposure to the primary outbreak case and no direct exposure to the outbreak site during the incubation period.

**Close contacts** were individuals that either:

1) provided direct care for the case, including HCWs, family members or other caregivers, or who had other similar close physical contact without consistent and appropriate use of personal protective equipment (PPE), OR

2) lived with or otherwise had close prolonged contact (i.e., for more than 15 minutes and within two metres) with a case without consistent and appropriate use of PPE and not isolating OR

3) had direct contact with infectious body fluids of a case (e.g., was coughed or sneezed on) while not wearing recommended PPE.

**Incubation Period:** Time from exposure to an infection onset of symptoms was 14 days.

**Period of Communicability:** Period of time during which the sick person can transmit disease to others**.**

*For symptomatic cases*: 2 days prior to onset until 14 days post onset or symptom resolution, whichever is longer. Patients were isolated for 14 days or until symptom resolution.

*For asymptomatic cases:* 2 days prior to positive specimen collection date until 10 days after positive specimen collection date. If the case develops symptoms, until 10 days following symptom onset or symptom resolution, whichever is longer. Patients were isolated for 10 days after positive specimen collection date or longer if symptoms developed.

**Case-Control Data Collection Details**

Data collected included demographics (patient identifiers, names, date of birth, sex), Elixhauser Index and individual comorbidities[2], baseline laboratory measures within 24 hours of admission (i.e. white blood cell count, lymphocyte, creatinine, platelet, hemoglobin, neutrophils), Braden Scale for mobility subscales within 24 hours of admission as a surrogate for high needs patients who may be bedbound and requiring more hands-on care[3] , baseline medications from admission to index date for cases and controls (i.e. ACE inhibitors, angiotensin II inhibitors, angiotensin receptor blockers, neprilysin inhibitors, diuretics, immunosuppressive agents), hospitalization characteristics in the 7- days prior to index date for cases and controls (i.e. length of stay prior to outbreak exposure, transfers between and within units, days in single, double, or multi-bedded rooms [3 or more beds]).

**Baseline laboratory measures category definitions:**

Laboratory measures were categorized as either abnormal (high/low) or normal based on laboratory values falling outside normal ranges according to [laboratory reference ranges](https://www.albertahealthservices.ca/assets/wf/lab/wf-lab-cbc-reference-intervals-calgary.pdf) and clinical classification.

- White blood cell count (WBC): High values were classified as “abnormal”; normal and low values were categorized as “normal”
- Lymphocyte: Low values were classified as “abnormal”, high and normal values were categorized as “normal”
- Creatinine: High values were classified as “abnormal”, low and normal values were categorized as “normal”
- Hemoglobin: Low values were classified as “abnormal”, high and normal values were categorized as “normal”
- Neutrophil: High values were classified as “abnormal”, low and normal values were categorized as “normal”

**Immunosuppressive Agents:** azathioprine, methotrexate, mycophenolate mofetil, tracrolimus, vedolizumab

**Discharge Abstract Database:**  contains information on separations (including discharges, deaths, sign-outs and transfers) from acute care, adult inpatient rehabilitations, subacute care, and adult inpatient mental health institutions in Alberta. It includes clinical data (diagnoses, interventions), demographic data (sex, date of birth, postal code, residence code), administrative data (institution number, admission category, length of stay, disposition, inter-institutional transfers), and data used to evaluate patient length of stay and resource consumption (Case Mix Group Plus and Resource Intensity Weight).

**Admission, Discharge, and Transfer Database:** contains admission, discharge, and transfer data for services (inpatient, ambulatory, and seniors) attached to an acute care facility including changes of attending physicians, patients’ encounter records, and room/bed transfers.

**References**

1. O'Grady H, Dixit D, Khawaja Z, Snedeker K, Ellison J, Erebor J, et al. Asymptomatic Severe Acute Respiratory Syndrome Coronavirus-2 (SARS-CoV-2) Infection in Adults is Uncommon using Rigorous Symptom Characterization and Follow-up in an Acute Care Adult Hospital Outbreak. Infect Control Hosp Epidemiol. 2022(Online ahead of print):1-25. 10.1017/ice.2022.168.

2. van Walraven C, Austin PC, Jennings A, Quan H, Forster AJ. A modification of the Elixhauser comorbidity measures into a point system for hospital death using administrative data. Med Care. 2009;47(6):626-33. 10.1097/MLR.0b013e31819432e5.

3. Bergstrom N, Demuth PJ, Braden BJ. A clinical trial of the Braden Scale for Predicting Pressure Sore Risk. Nurs Clin North Am. 1987;22(2):417-28.
