# Supplementary material for: Patient and ward related risk factors in a multi-ward nosocomial outbreak of COVID-19: Outbreak investigation and matched case–control study
Source: Antimicrob Resist Infect Control. 2023 Mar 22;12:21. doi: 10.1186/s13756-023-01215-1 (PMC10031162; doi:10.1186/s13756-023-01215-1)
Supplement: Supplementary file 2 — Additional file 2. Environmental sampling results: Includes three tables of the environmental sampling results before and after cleaning, and cultivatable virus detected from clinical and environmental specimens. [file 13756_2023_1215_MOESM2_ESM.docx]

**ADDITIONAL FILE 2: ENVIRONMENTAL SAMPLING RESULTS**

**Table A1. Detection of SARS-CoV-2 by RT-PCR by environmental swabbing on Ward A pre- and post-cleaning by Environmental Services**

| **Ward A Pre-Clean** | | | | | | | | | |
| --- | --- | --- | --- | --- | --- | --- | --- | --- | --- |
| **Location** | **RT-PCR result** | **E/MS Duplex E Gene Ct** | | | **CDC N2** | | | |  |
| nursing desk computer keyboard | negative | neg |  |  | neg | neg | neg |  |  |
| room 33 bathroom floor | negative | neg |  |  | neg | neg | neg |  |  |
| room 3 toilet seat | negative | neg |  |  | neg | neg | neg |  |  |
| room 3 call  bell | negative | neg |  |  | neg | neg | neg |  |  |
| room 3 underneath toilet seat | negative | neg |  |  | neg | neg | neg |  |  |
| NSG station telemetry keyboard | negative | neg |  |  | neg | neg | neg |  |  |
| nursing station call bell | negative | neg |  |  | neg | neg | neg |  |  |
| nursing station patient chart | negative | neg |  |  | neg | neg | neg |  |  |
| family room telephone | negative | neg |  |  | neg | neg | neg |  |  |
| room 3 sharps container | negative | neg |  |  | neg | neg | neg |  |  |
| **room 3 white board** | **positive** | neg | **38.19** | neg | neg | neg | **37.43** |  |  |
| room 3 blood pressure cuff | negative | neg |  |  | neg | neg | neg |  |  |
| room 3 vital signs machine | negative | neg |  |  | neg | neg | neg |  |  |
| room 3 sink | negative | neg |  |  | neg | neg | neg |  |  |
| room 3 bed siderail | negative | neg |  |  | neg | neg | neg |  |  |
| room 3 keyboard | negative | neg |  |  | neg | neg | neg |  |  |
| room 3 whiteboard markers | negative | neg |  |  | neg | neg | neg |  |  |
| kitchen | negative | neg |  |  | neg | neg | neg |  |  |
| room 18-3 oxygen device | negative | neg |  |  | neg | neg | neg |  |  |
| room 18-4 sharps container | negative | neg |  |  | neg | neg | neg |  |  |
| room 18-1 sink | negative | neg |  |  | neg | neg | neg |  |  |
| room 18 glove box - small | negative | neg |  |  | neg | neg | neg |  |  |
| room 18-1 curtain | negative | neg |  |  | neg | neg | neg |  |  |
| room 17-18 bathroom call bell | negative | neg |  |  | neg | neg | neg |  |  |
| room 17-18 bathroom toilet seat | negative | neg |  |  | neg | neg | neg |  |  |
| **room 17-18 bathroom commode chair** | **positive** | neg | neg | neg | neg | neg | **37.38** |  |  |
| nursing station | negative | neg |  |  | neg | neg | neg |  |  |
| **room 17-2 call bell** | **positive** | **30.2** |  |  | **28.75** | **28.78** | **28.85** |  |  |
| room 17-18 bathroom sink taps | negative | neg |  |  | neg | neg | neg |  |  |
| room 17-18 bathroom stall door | negative | neg |  |  | neg | neg | neg |  |  |
| room 17-18 bathroom toilet seat | negative | neg |  |  | neg | neg | neg |  |  |
| room 17-18 bathroom toilet paper holder | negative | neg |  |  | neg | neg | neg |  |  |
| nursing station call bell | negative | neg |  |  | neg | neg | neg |  |  |
| room 17-1 light switch | negative | neg |  |  | neg | neg | neg |  |  |
| room 16-3 call bell | negative | neg |  |  | neg | neg | neg |  |  |
| room 17 light switch | negative | neg |  |  | neg | neg | neg |  |  |
| room 17 bathroom glove box | negative | neg |  |  | neg | neg | neg |  |  |
| **room 16-3 window ledge** | **positive** | **35.9** |  |  | **35.75** | **36.35** | **35.63** |  |  |
| room 16-3 call bell | negative | neg |  |  | neg | neg | neg |  |  |
| **room 16-3 bathroom toilet paper dispenser** | **positive** | **37.88** |  |  | neg | neg | neg |  |  |
| Room 16-3 call bell cancel button | negative | neg |  |  | neg | neg | neg |  |  |
| **room 16-3 curtains** | **positive** | **36.37** |  |  | **35.84** | **34.32** | **36.68** |  |  |
| room 16-3 suction supplies | negative | neg |  |  | neg | neg | neg |  |  |
| room 17-4 bed rails | negative | neg |  |  | neg | neg | neg |  |  |
| room 17-4 paper tower dispenser | negative | neg |  |  | neg | neg | neg |  |  |
| room 17 dry erase marker | negative | neg |  |  | neg | neg | neg |  |  |
| **room 17 bed side table** | **positive** | neg | **38.45** | neg | **37.42** | neg | neg |  |  |
| room 17-1 call bell | negative | neg |  |  | neg | neg | neg |  |  |
| **room 16 bathroom sink taps** | **positive** | neg | neg | **38.13** | neg | **36.68** | neg |  |  |
| room 17 bedside drawers | negative | neg |  |  | neg | neg | neg |  |  |
| room 16 toilet bowl lip | negative | neg |  |  | neg | neg | neg |  |  |
| room 17-3 window ledge | negative | neg |  |  | neg | neg | neg |  |  |
| room 3 bathroom toilet flush handle | negative | neg |  |  | neg | neg | neg |  |  |
| Room 18-3 dry erase marker | negative | neg |  |  | neg | neg | neg |  |  |
| room 18-3 window ledge | negative | neg |  |  | neg | neg | neg |  |  |
| room 18-4 call bell | negative | neg |  |  | neg | neg | neg |  |  |
| room 18-3 call bell | negative | neg |  |  | neg | neg | neg |  |  |
| room 18-4 wall | negative | neg |  |  | neg | neg | neg |  |  |
| nursing station central monitor | negative | neg | neg | neg | neg | neg | neg |  |  |
| room 20 TV remote | negative | neg | neg | neg | neg | neg | neg |  |  |
| room 20 fridge handle | **positive** | **43.02** | **40.63** | neg | **36.09** | **35.12** | **37.42** |  |  |
| tele cables | negative | neg | neg | neg | neg | neg | neg |  |  |
| tele pack #537 | negative | neg | neg | neg | neg | neg | neg |  |  |
| tele cables | negative | neg | neg | neg | neg | neg | neg |  |  |
| room 20 key pad and door handle | negative | neg | neg | neg | neg | neg | neg |  |  |
| room 20 staff room microwave | negative | neg | neg | neg | neg | neg | neg |  |  |
| tele pack #663 | negative | neg | neg | neg | neg | neg | neg |  |  |
| room 20 couch arm | negative | neg | neg | neg | neg | neg | neg |  |  |
| room 21 staff lockers | negative | neg | neg | neg | neg | neg | neg |  |  |
| Wimed #7 SPO2 probe | negative | neg | neg | neg | neg | neg | neg |  |  |
| room 2 SPO2 probe | **positive** | **28.92** | **28.72** | **28.68** | **27.43** | **27.52** | **27.47** |  |  |
| room 2 thermometer | **positive** | **35.38** | **34.71** | **34.85** | **33.52** | **33.81** | **33.96** |  |  |
| room 2 O2 dial | negative | neg | neg | neg | neg | neg | neg |  |  |

| **Ward A Post-Clean** | | | | | | | |
| --- | --- | --- | --- | --- | --- | --- | --- |
| **Location** | **RT-PCR result** | **E/MS Duplex**  **E Gene Ct** | | | **CDC N2** | | |
| room 15 bathroom door handle | negative | neg |  |  | neg | neg | neg |
| room 16B overhead bed table | negative | neg |  |  | neg | neg | neg |
| room 2 SPO2 probe | **positive** | **35.88** | **37.08** | **37.89** | **36.4** | neg | neg |
| room 17/18 bathroom sink handles | negative | neg |  |  | neg | neg | neg |
| room 17/18bathroom toilet | negative | neg |  |  | neg | neg | neg |
| room 17C window ledge | negative | neg |  |  | neg | neg | neg |
| room 17B call bell | negative | neg |  |  | neg | neg | neg |
| wimed#8 keyboard | negative | neg |  |  | neg | neg | neg |
| wimed #6 thermometer | negative | neg |  |  | neg | neg | neg |
| wimed #1 O2 probe | negative | neg |  |  | neg | neg | neg |

**Table A2. Detection of SARS-CoV-2 by RT-PCR by environmental swabbing on Ward B**

| **Ward B** | | | | | | | |
| --- | --- | --- | --- | --- | --- | --- | --- |
| **Location** | **RT-PCR result** | **E/MS Duplex E Gene Ct** | | | **CDC N2** | | |
| hallway patient education papers | negative | neg |  |  | neg | neg | neg |
| med room | negative | neg |  |  | neg | neg | neg |
| room 22 curtain | negative | neg |  |  | neg | neg | neg |
| room 22 wimed #7 O2 sat probe | negative | neg |  |  | neg | neg | neg |
| med room fridge door handle | negative | neg |  |  | neg | neg | neg |
| room 23 (covid pos) bathroom stall door | negative | neg |  |  | neg | neg | neg |
| room 24 bathroom inside door handle | negative | neg |  |  | neg | neg | neg |
| main desk main counter by unit clerk, public side | negative | neg |  |  | neg | neg | neg |
| room 24 locker handles | negative | neg |  |  | neg | neg | neg |
| room 23 (covid pos) bathroom taps | negative | neg |  |  | neg | neg | neg |
| photocopier | negative | neg |  |  | neg | neg | neg |
| Room 23 (covid pos) bedrail | negative | neg |  |  | neg | neg | neg |
| main desk phone | negative | neg |  |  | neg | neg | neg |
| kitchen code box (door) | negative | neg |  |  | neg | neg | neg |
| staff room phone | negative | neg |  |  | neg | neg | neg |
| room 25 staff washroom door knob | negative | neg |  |  | neg | neg | neg |
| nursing station tape dispenser | negative | neg |  |  | neg | neg | neg |
| room 22 wimed cavet(?) #1 | negative | neg |  |  | neg | neg | neg |
| transport monitor #5 | negative | neg |  |  | neg | neg | neg |
| staff room paper towel dispenser | **positive** | neg | neg | neg | neg | neg | **36.28** |
| staff room microwave | negative | neg |  |  | neg | neg | neg |
| staff room fridge door handle | negative | neg |  |  | neg | neg | neg |
| staff room door lock keypad | negative | neg |  |  | neg | neg | neg |
| room 24 side table | negative | neg |  |  | neg | neg | neg |
| room 24 WMHS(?) | negative | neg |  |  | neg | neg | neg |
| main desk central monitor CIC #2 | negative | neg |  |  | neg | neg | neg |
| room 22 call bell | negative | neg |  |  | neg | neg | neg |
| tPack 633 | negative | neg |  |  | neg | neg | neg |
| main desk pt chart (from covid pos pt) | negative | neg |  |  | neg | neg | neg |
| main desk MPR slot | negative | neg |  |  | neg | neg | neg |
| front desk call system | negative | neg |  |  | neg | neg | neg |
| room 23 (covid pos) O2 sat probe | negative | neg |  |  | neg | neg | neg |
| room 23 bathroom  toilet | negative | neg |  |  | neg | neg | neg |
| room 23 whiteboard eraser | **positive** | neg | neg | neg | **35.75** | neg | neg |
| room 24 call bell | negative | neg |  |  | neg | neg | neg |
| room 22 thermometer | negative | neg |  |  | neg | neg | neg |
| room 22 transfer belt | negative | neg |  |  | neg | neg | neg |
| room 22 side table | negative | neg |  |  | neg | neg | neg |
| med room med drawers | negative | neg |  |  | neg | neg | neg |
| room 24 lightswitch | negative | neg |  |  | neg | neg | neg |
| room 24 whiteboard | negative | neg |  |  | neg | neg | neg |
| room 24 sink taps | negative | neg |  |  | neg | neg | neg |
| room 23 (covid pos) commode arms | negative | neg |  |  | neg | neg | neg |
| room 23 (covid pos) call bell | negative | neg |  |  | neg | neg | neg |
| room 23 (covid pos) bathroom door handle | negative | neg |  |  | neg | neg | neg |
| room 23(covid pos) window sill | **positive** | neg | neg | neg | **37.91** | **37.42** | **37.17** |
| room 23 (covid pos) toilet paper dispenser | negative | neg |  |  | neg | neg | neg |
| room 22 wimed #1 mouse | negative | neg |  |  | neg | neg | neg |
| BS1- Ward B bladder scanner wand | Neg |  |  |  |  |  |  |
| BS2- Ward B tabletop | Neg |  |  |  |  |  |  |
| BS3- Ward B screen and buttons | Neg |  |  |  |  |  |  |
| BS4- Ward B screen handle | Neg |  |  |  |  |  |  |
| BS5- Ward B aquasonic gel dispenser bottle | Neg |  |  |  |  |  |  |
| BS6- Ward B table top handle | Neg |  |  |  |  |  |  |
| BS7- Ward B gel bottle spout | Neg |  |  |  |  |  |  |

**Table A3. Cultivatable SARS-CoV-2 from clinical and environmental specimens from consenting symptomatic patients on Wards A and B ***

| **Patient** | **Ct value at Collection** | **Specimen** | **Cultivatable Virus (pfu/ml)** |
| --- | --- | --- | --- |
| Patient 1 – Ward A | 18.7 (E gene)  16.4 (N gene) | nasopharyngeal swab | 3.55 x 10^3^ |
|  |  | cough bag | 7.45x10^2^ |
|  |  | left and right hands bag wash (random) | 2.30x10^2^ |
|  |  | speak bag | Not detected |
|  |  | kiss bag | 3.50x10^1^ |
|  |  | discarded facial tissue | 6.35x10^2^ |
|  |  | cell phone | 1.95x10^2^ |
|  |  | baseline saliva sample | 2.30x 10^3^ |
|  |  | replicate saliva sample | 2.05x10^3^ |
|  |  | saliva in petri dish in bathroom (dried) | 1.45x10^4^ |
|  |  | saliva in petri dish in room at 2 hrs (dried) | 1.50x10^3^ |
|  |  | saliva in petri dish in room at 2 hours (dried) | 8.50 x10^2^ |
|  |  | replicate saliva baseline sample | 1.00 x10^3^ |
|  |  | saliva in petri dish in bathroom at 2 hours DMEM | 8.00 x10^2^ |
|  |  | saliva in petri dish in room at 2 hours DMEM | 1.25 x10^3^ |
|  |  | saliva in petri dish in room at 2 hours DMEM | 9.00x10^2^ |
| Patient 2 Ward A | N/A (E gene)  11.4 (N gene) | nasopharyngeal swab | 2.70x 10^3^ |
| Patient 3 Ward A | 16.7 (E gene)  13.5 (N gene) | nasopharyngeal swab | 5.05x10^4^ |
|  |  | Saliva | Not detected |
|  |  | cough bag | Not detected |
|  |  | speak bag | Not detected |
|  |  | hand bag (random) | Not detected |
| Patient 4 – Ward A† | 19.9 (E gene)  14.1 (N gene) | nasopharyngeal swab | 2.3 x 10^3^ |
|  |  | Saliva | 2.45x10^3^ |
|  |  | cell phone | Not detected |
|  |  | drool facecloth lying on bed | 1.15x10^2^ |
| Patient 5 – Ward B |  | nasopharyngeal swab | 9.00x10^3^ |
|  |  | saliva - with lots of water | 3.00x10^2^ |
|  |  | cough bag | Not detected |
|  |  | speak bag | Not detected |
|  |  | –kiss sample | Not detected |
|  |  | right hand bag wash (random) | 9.50x10^1^ |
| Patient 6-Ward B | 22.9 (E gene)  19.2(N gene) | nasopharyngeal swab | 2.70x10^4^ |
|  |  | saliva | 1.1x10^3^ |
|  |  | cough bag | Not detected |
|  |  | hand bag wash (random) | Not detected |
|  |  | kiss specimen | Not detected |
|  |  |  |  |
| Patient 7 – Ward B | 23.7(E gene) 18.6 (N gene) | throat swab | 4.00x10^3^ |
|  |  | kiss specimen | Not detected |
|  |  | saliva | 4.00x10^2^ |
|  |  | speak bag | Not detected |
|  |  | sputum | 5.20x10^5^ |
|  |  | call bell | Not detected |
|  |  | cell phone | Not detected |
|  |  | dentures at bedside | Not detected |
|  |  | post nose blow on gauze | 2.20x10^4^ |
|  |  | right hand | 3.00x10^2^ |
|  |  | left hand shake (post cleaning) from right hand | 1.40x10^2^ |
|  |  | pulse oximeter | Not detected |
|  |  | gloved hand of HCW | Not detected |
| Patient 8 – Ward B | 15.8(E gene)  12.2 (N gene) | nasopharyngeal swab | 2.50x10^5^ |
|  |  | saliva swab | 6.00 x 10^1^ |
|  |  | saliva ( <0.5 ml) | Not detected |
|  |  | cough bag –(very weak cough) | Not detected |
|  |  | kiss bag | Not detected |
|  |  | hands | Not detected |
|  |  | cell phone | Not detected |
|  |  | pulse oximeter | Not detected |
|  |  | nasal prongs | 5.0 x 10^0^ |
|  |  | bed rail | Not detected |
| Comments |  | †Collected in ICU shortly after transfer from Ward A | |

*These data were included in aggregate in a study by Lin et al. ^11^
