# Supplementary material for: Patient and ward related risk factors in a multi-ward nosocomial outbreak of COVID-19: Outbreak investigation and matched case–control study
Source: Antimicrob Resist Infect Control. 2023 Mar 22;12:21. doi: 10.1186/s13756-023-01215-1 (PMC10031162; doi:10.1186/s13756-023-01215-1)
Supplement: Supplementary file 3 — Additional file 3. Additional case-control details and results: Includes case-control matching details, breakdown of comorbidities between cases and matched controls, and independent risk factors for nosocomial COVID-19 using different cut-off points for the percent exposure on multi-bedded rooms. [file 13756_2023_1215_MOESM3_ESM.docx]

**ADDITIONAL FILE 3: ADDITIONAL CASE-CONTROL DETAILS AND RESULTS**

**Case-Control Matching Details**

The case control study included all 39 case patients matched to 183 controls, of which 74 were unique control patients. Of the 74 unique control patients, 38 (51.3%) were matched only once to a case, 10 (13.5%) were matched twice to cases, eight (10.8%) were matched three times, nine (12.2%) were matched four times, and nine (12.2%) were matched five or more times. Controls were randomly selected for cases with more than five matched controls.

**Table A4: Comorbidities among Nosocomial COVID-19 Cases and Matched Controls**

| **Variables** | **Nosocomial COVID-19 N=39 (%)** | **Matched control N=70 (%)** | **95%CI and P value** |
| --- | --- | --- | --- |
| AIDS | 0 (0.0%) | 0 (0.0%) | N/A |
| Anemia, Blood Loss | 0 (0.0%) | 0 (0.0%) | N/A |
| Anemia, Deficiency | 2 (5.1%) | 5 (7.1%) | 0.82, 0.732 |
| Alcohol | 3 (7.7%) | 3 (4.3%) | 1.59, 0.290 |
| Cardiac Arrhythmias | 14 (35.9%) | 26 (37.1%) | 1.03, 0.887 |
| Coagulopathy | 4 (10.3%) | 6 (8.6%) | 1.03, 0.934 |
| Congestive heart failure | 15 (38.5%) | 24 (34.3%) | 1.08, 0.748 |
| Chronic Pulmonary Disorders | 2 (5.1%) | 6 (8.6%) | 0.56, 0.376 |
| Depression | 2 (5.1%) | 2 (2.9%) | 1.37, 0.546 |
| Diabetes, uncomplicated | 2 (5.1%) | 5 (7.1%) | 0.73, 0.513 |
| Diabetes, complicated | 7 (18.0%) | 16 (22.9%) | 0.67, 0.16 |
| Drug abuse | 0 (0.0%) | 2 (2.9%) | N/A |
| Fluid/Electrolyte Disorders | 14 (35.9%) | 10 (14.3%) | 3.16, 0.001 |
| Hypertension, uncomplicated | 10 (25.6%) | 21 (30.0%) | 0.75, 0.272 |
| Hypertension, complicated | 0 (0.0%) | 1 (1.4%) | N/A |
| Hypothyroidism | 2 (5.1%) | 1 (1.4%) | 3.31, 0.297 |
| Liver Disease | 2 (5.1%) | 2 (2.9%) | 1.94, 0.245 |
| Lymphoma | 0 (0.0%) | 1 (1.4%) | N/A |
| Metastatic cancer | 0 (0.0%) | 2 (2.9%) | N/A |
| Obesity | 1 (2.6%) | 1 (1.4%) | 1.68, 0.586 |
| Other Neurological Disorders | 4 (10.3%) | 2 (2.9%) | 2.04, 0.020 |
| Paralysis | 0 (0.0%) | 1 (1.4%) | N/A |
| Peptic Ulcer Disease | 0 (0.0%) | 0 (0.0%) | N/A |
| Peripheral Vascular Disorders | 1 (2.6%) | 1 (1.4%) | 1.26, 0.724 |
| Psychoses | 0 (0.0%) | 0 (0.0%) | N/A |
| Pulmonary Circulation Disorders | 3 (7.7%) | 3 (4.3%) | 1.25, 0.581 |
| Renal failure | 3 (7.7%) | 4 (5.7%) | 1.48, 0.415 |
| Rheumatoid Arthritis | 0 (0.0%) | 2 (2.9%) | N/A |
| Solid tumor | 2 (5.1%) | 5 (7.1%) | 0.74, 0.569 |
| Valvular Disease | 4 (10.3%) | 11 (15.7%) | 0.76, 0.523 |
| Weight Loss | 2 (5.1%) | 5 (7.1%) | 0.65, 0.334 |

**Table A5. Independent risk factors for nosocomial COVID-19 (25% cut-off point)**

| **Variables** | **IRR** | **95%CI** | **P value** |
| --- | --- | --- | --- |
| Age | 1.04 | 1.00-1.08 | 0.080 |
| Underlying Disease |  |  |  |
| Fluid/Electrolyte Disorders | 3.61 | 1.50-8.71 | 0.004 |
| Other Neurological Disorders | 2.44 | 1.23-4.87 | 0.011 |
| Medications |  |  |  |
| Days on diuretic | 1.05 | 0.95-1.15 | 0.344 |
| Days on immunosuppressive agents | 1.34 | 1.03-1.76 | 0.031 |
| Elixhauser score (AHRQ) | 0.97 | 0.93-1.01 | 0.137 |
| Abnormal Lymphocyte | 1.39 | 0.75-2.58 | 0.302 |
| Percent of Exposure on Multi-bed Room |  |  |  |
| 0-25% of time | Reference | Reference | Reference |
| >25% of time | 1.89 | 1.04-3.43 | 0.036 |

IRR= incidence rate ratio; CI=confidence interval

**Table A6. Independent risk factors for nosocomial COVID-19 (75% cut-off point)**

| **Variables** | **IRR** | **95%CI** | **P value** |
| --- | --- | --- | --- |
| Age | 1.04 | 0.99-1.08 | 0.114 |
| Underlying Disease |  |  |  |
| Fluid/Electrolyte Disorders | 4.01 | 1.74-9.20 | 0.001 |
| Other Neurological Disorders | 3.02 | 1.45-6.27 | 0.003 |
| Medications |  |  |  |
| Days on diuretic | 1.08 | 0.98-1.18 | 0.126 |
| Days on immunosuppressive agents | 1.39 | 1.06-1.83 | 0.018 |
| Elixhauser score (AHRQ) | 0.97 | 0.93-1.01 | 0.115 |
| Abnormal Lymphocyte | 1.17 | 0.63-2.17 | 0.616 |
| Percent of Exposure on Multi-bed Room |  |  |  |
| 0-75% of time | Reference | Reference | Reference |
| >75% of time | 3.32 | 1.47-7.02 | 0.003 |

IRR= incidence rate ratio; CI=confidence interval
